# Real-world patterns of immunoglobulin replacement therapy for infection prevention in common variable immunodeficiency: a multicenter nationwide study

**DOI:** 10.3389/fimmu.2025.1640290

**Published:** 2025-07-23

**Authors:** Pedro Moral Moral, Victor Garcia-Bustos, Héctor Balastegui-Martin, Sandra Martínez Mercader, Carmen Bracke, Lourdes Mateu, Xavier Solanich, Arnau Antolí, Juan Luis Carrillo-Linares, Ángel Robles-Marhuenda, Francesc Puchades, Ana Pelaez Ballesta, Nuria López-Osle, Miguel Ángel Torralba-Cabeza, Ana María Bielsa Masdeu, Jorge Gil Niño, Nuria Tornador Gaya, Guillem Pascual Castellanos, Rosario Sánchez-Martínez, José Manuel Barragán-Casas, Andrés González-García, José Luís Patier de la Peña, Daniel López-Wolf, Antonia Mora Rufete, Alba Canovas Mora, Marta Dafne Cabañero-Navalon

**Affiliations:** ^1^ Primary Immunodeficiencies Unit, Department of Internal Medicine, University and Polytechnic Hospital La Fe, Valencia, Spain; ^2^ Research Group of Chronic Diseases and HIV Infection, Health Research Institute La Fe, Valencia, Spain; ^3^ Severe Infection Research Group, Health Research Institute La Fe, Valencia, Spain; ^4^ Infectious Diseases Service, Germans Trias i Pujol Hospital, Badalona, Spain; ^5^ Fight Infections Foundation, Germans Trias i Pujol Hospital, Badalona, Spain; ^6^ Adult Primary Immunodefciency Unit (UFIPA), Internal Medicine Department, Bellvitge University Hospital, L’Hospitalet de Llobregat, Barcelona, Spain; ^7^ The Systemic, Vascular Diseases and Ageing Group, Bellvitge Biomedical Research Institute (IDIBELL), L’Hospitalet de Llobregat, Barcelona, Spain; ^8^ Clinical Sciences Department, Faculty of Medicine and Health Sciences, University of Barcelona, Barcelona, Spain; ^9^ Department of Internal Medicine, Virgen de la Victoria University Hospital, Málaga, Spain; ^10^ Department of Internal Medicine, La Paz University Hospital, Madrid, Spain; ^11^ Department of Internal Medicine, University General Hospital of Valencia, Valencia, Spain; ^12^ Department of Internal Medicine, Rafael Méndez University Hospital, Murcia, Spain; ^13^ Department of Internal Medicine, Cruces University Hospital, Bizkaia, Spain; ^14^ Unit for Rare Diseases, Internal Medicine Service, Lozano Blesa University Hospital, Zaragoza, Spain; ^15^ Department of Internal Medicine, Miguel Servet University Hospital, Zaragoza, Spain; ^16^ Immunodeficiencies Clinic, Internal Medicine Department, 12 de Octubre Hospital, Madrid, Spain; ^17^ Department of Internal Medicine, University General Hospital of Castellón, Castellón, Spain; ^18^ Internal Medicine Department, Dr. Balmis General University Hospital, Alicante Institute for Health and Biomedical Research, Alicante, Spain; ^19^ Department of Internal Medicine, Complejo Asistencial de Ávila, Ávila, Spain; ^20^ Systemic Autoimmune Diseases Unit, Internal Medicine Service, Ramón y Cajal Hospital, Ramón y Cajal Health Research Institute, Madrid, Spain; ^21^ Department of Internal Medicine, University Hospital Alcorcón Foundation, Madrid, Spain; ^22^ Department of Internal Medicine, General University Hospital of Elche, Alicante, Spain

**Keywords:** common variable immunodeficiency (CVID), immunoglobulin replacement therapy (IGRT), subcutaneous immunoglobulin (SCIg), intravenous immunoglobulin (IVIg), real-world evidence

## Abstract

**Background and aims:**

Immunoglobulin replacement therapy (IgRT), administered intravenously (IVIg) or subcutaneously (SCIg), is the cornerstone treatment for patients with Common Variable Immunodeficiency (CVID). Although both modalities demonstrate similar efficacy, SCIg is associated with fewer systemic adverse events and increased patient autonomy. Despite these advantages, its utilization remains limited in certain regions, particularly in the Mediterranean region. This study aimed to evaluate real-world patterns of IgRT use in Spanish CVID patients and provide a comprehensive analysis of the factors associated with IVIg and SCIg administration in routine clinical practice.

**Methods:**

A cross-sectional, multicenter study was conducted using data from the GTEM-SEMI-CVID Registry, encompassing 212 adult CVID patients receiving IgRT across Spain. Patients were grouped based on the administration route: IVIg and SCIg. Demographic, clinical, and immunological data, including IgRT modality, dosage, administration setting, and comorbidities, were collected. Comparative statistical analyses were performed to identify differences between both treatment groups.

**Results:**

Of the 212 patients, 58.5% received IVIg and 41.5% received SCIg. SCIg recipients were younger (47.5 vs. 54.8 years, *p* = 0.003) and predominantly treated at home (80.6% *vs*. 1.6%, *p* < 0.001), compared to those receiving IVIg. SCIg use was significantly higher in tertiary hospitals compared to secondary ones (44.4% *vs*. 17.4%, *p* = 0.0136). Infection rates, autoimmune comorbidities, weekly doses (7.2 g for IVIg *vs*. 7.7 g for SCIg, *p* = 0.142), and IgG trough levels were comparable across groups.

**Conclusion:**

This study provides real-world evidence on IgRT patterns in Spanish patients with CVID, revealing a marked increase in SCIg use over the past decade, although IVIg remains predominant, especially in secondary hospitals. Age significantly influenced the choice of modality, with IVIg preferred for older patients and SCIg for younger ones, while disease severity did not impact this decision. These findings underscore the need to optimize access to SCIg, particularly in secondary centers, to enhance patient autonomy and improve therapeutic outcomes.

## 1 Introduction

Immunoglobulin replacement therapy (IgRT), administered either intravenously (IVIg) or subcutaneously (SCIg), is a cornerstone in the management of patients with Inborn Errors of Immunity (IEI), concretely those characterized by impaired antibody production (1). IgRT has proven effective in reducing infection frequency and severity, preventing organ damage, and improving survival rates. In Spain, IVIg has been widely used since the 1980s, with 5% and 10% formulations commercially available. SCIg 20% was introduced in the early 2000s and gradually implemented in specialized centers. Facilitated SCIg (fSCIg) with recombinant human hyaluronidase at 10% concentration became available around 2017, broadening outpatient administration options.

While both IVIg and SCIg are considered equally effective (2), SCIg is associated with fewer systemic adverse events, enhanced quality of life, and greater patient autonomy (2, 3). Although the acquisition cost per gram is higher for SCIg (approximately €66.10/g) compared to IVIg (€46.80/g), SCIg has been shown to be more cost-effective in the long term (4, 5). This is largely due to the reduction in indirect healthcare costs, as SCIg avoids the need for regular hospital day-care unit visits, dedicated infusion chairs, and nursing supervision at each administration. Despite the potential cost savings of SCIg, its use depends on several factors, including institutional experience, availability of trained nursing staff, and structured care pathways. In some centers, limited infrastructure or familiarity may restrict its use. Ultimately, treatment decisions are made through shared decision-making, balancing clinical suitability, patient preferences, and logistical feasibility.

In Europe, the clinical use of IgRT has been extensively mapped by the Primary Immunodeficiency Care Working Party of the European Society for Immunodeficiencies (ESID) through the European Immunoglobulin Map project (6). This pan-European survey, initiated in 2006, tracks the evolving use of Ig products across European countries. By 2014, data from 35 countries revealed that IVIg was universally available, whereas access to SCIg varied significantly. Scandinavian countries and parts of Central Europe, such as Belgium, Norway, Sweden, and the United Kingdom, reported widespread use of SCIg, particularly in pediatric populations, where it is often administered as home-based therapy (7). In contrast, Mediterranean countries showed considerably lower rates of SCIg utilization, with outdated reported usage barely reaching 15% in Spain in 2014 (6).

Despite its clinical and economic advantages, SCIg remains underutilized in Spain, especially among adult patients. Furthermore, since the last ESID survey in 2014, there is a lack of updated, real-world data reflecting current IVIg and SCIg usage trends in Mediterranean countries such as Spain. Additionally, real-life data on specific prescription patterns to patient subgroups according to the severity of the phenotype or clinical course are scarce. This gap in knowledge hinders the ability to assess whether improvements in access and patient uptake have occurred over the past decade. Barriers such as limited awareness among healthcare professionals, economic constraints, and regional disparities in treatment availability may contribute to this underutilization (8). Addressing these limitations is crucial to homogeneously optimizing IgRT for patients with IEI.

In this context, the present study aims to assess the real-world use of IVIg and SCIg in patients with Common Variable Immunodeficiency (CVID), the most common symptomatic primary immunodeficiency (9). It seeks to identify the clinical and immunological factors influencing the choice of IgRT modality from a multicentric nationwide cohort of 250 adult CVID patients from tertiary and secondary hospitals in Spain, providing a comprehensive analysis of the factors associated with IVIg and SCIg administration in routine clinical practice.

## 2 Materials and methods

### 2.1 Study design

This study is a cross-sectional, multicenter analysis designed to evaluate the use of IgRT in patients with CVID in Spain. Data were extracted from the GTEM-SEMI-CVID Registry, a national multicenter registry established in 2019 by the Primary Immunodeficiency Unit at the University and Polytechnic Hospital La Fe, in collaboration with the Working Group on Rare Diseases (GTEM) of the Spanish Society of Internal Medicine (SEMI). This registry, which includes 17 Spanish centers, systematically collects clinical, immunological, and therapeutic information on CVID patients, as previously published (10).

For the present study, all patients aged ≥16 years with a confirmed diagnosis of CVID, according to the European Society for Immunodeficiencies (ESID) criteria (11), were considered eligible. Only patients who were actively receiving IgRT at the time of inclusion (between 2019 and 2022) were selected, and were classified into two groups according to the route of administration: IVIg or SCIg. A comparative analysis was performed to identify regional, clinical and immunological differences between both treatment modalities, alongside a detailed characterization of patient demographics, comorbidities, and therapeutic regimens associated with each IgRT approach.

**Figure 1:**
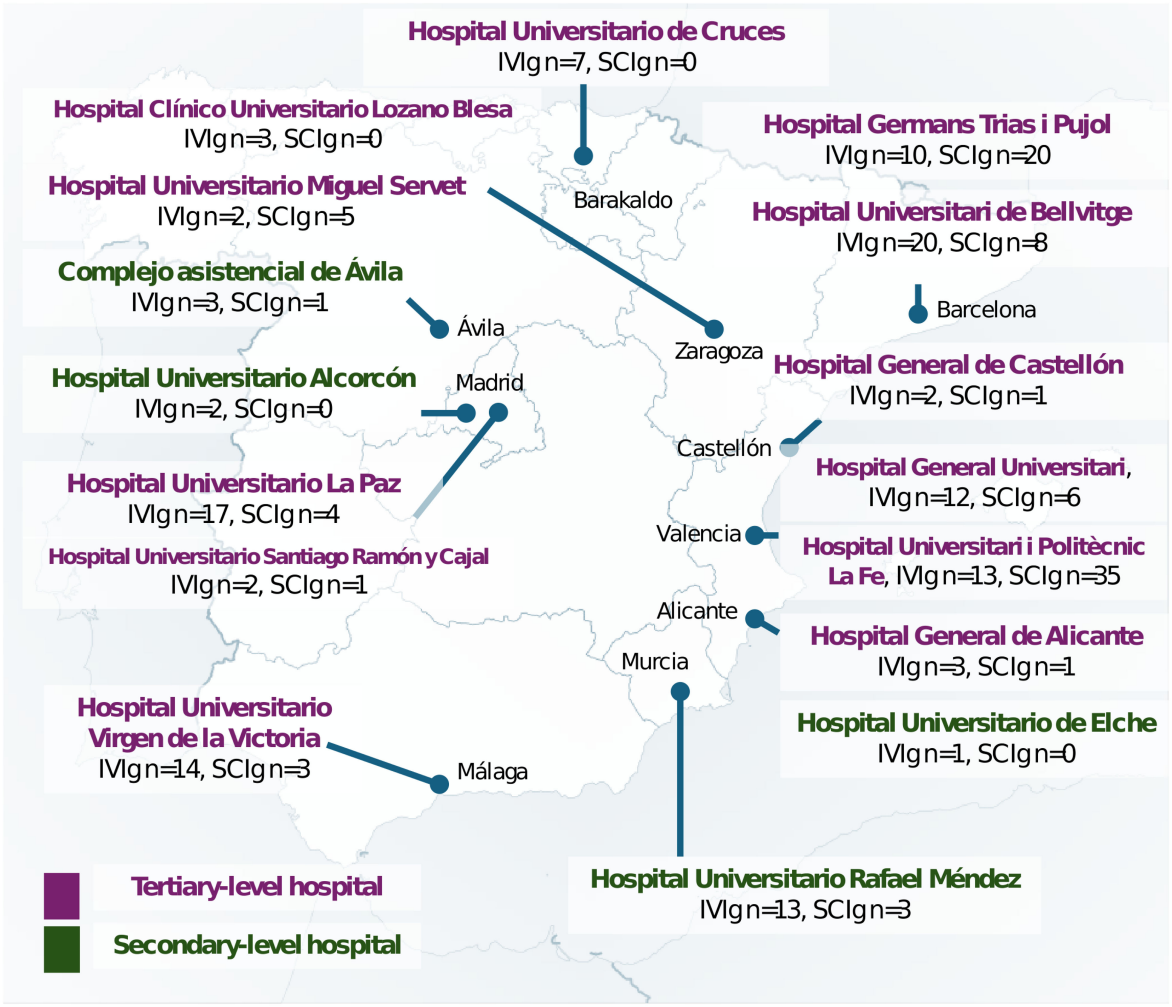
Spanish distribution of intravenous immunoglobulin therapy (IVIg) and subcutaneous immunoglobulin therapy (SCIg) according to the hospital level of care.

Information gathered included sociodemographic characteristics, IgRT details—such as treatment status, route of administration, formulation type, administration setting (hospital-based or home-based), weekly dose in grams, and frequency of administration (weekly, biweekly, every three weeks, or monthly)—and clinical history. Clinical variables included infectious and non-infectious comorbidities, immunological parameters, imaging findings, lung function tests, histopathological results, and the use of immunosuppressants. Additionally, the type of IgRT administered, IVIg or SCIg, was analyzed according to the level of care of the participating hospitals, categorized as either tertiary or secondary centers. In the Spanish healthcare system, tertiary hospitals are high-complexity referral centers offering specialized care and advanced diagnostic and therapeutic services, while secondary hospitals provide general specialist care to a defined population within a regional scope. Moreover, the geographical distribution of these centers across Spain was evaluated to identify potential regional variations in IgRT practices (**Figure 1**).

The infectious history of the patients comprised major bacterial infections, including pneumonia, meningitis or encephalitis, osteomyelitis, cellulitis, febrile urinary tract infections, sepsis of any origin, and opportunistic infections. Non-infectious comorbidities collected the actual or previous history of systemic autoimmune diseases, immune-mediated cytopenias, lymphadenopathy, splenomegaly, pulmonary involvement (including histologically-confirmed granulomatous-lymphocytic interstitial lung disease -GLILD- and bronchiectasis), enteropathy (non-infectious chronic diarrhea and malabsorption), hepatic manifestations (hepatomegaly and portal hypertension), immune-mediated skin disease, immune-mediated neurological disorder, and both solid and hematologic malignancies. Further information on the variable definitions can be found in Cabañero-Navalon (10).

Cardiovascular risk factors and events were also recorded, namely hypertension, dyslipidemia, type 1 and 2 diabetes mellitus, heart failure, chronic cerebrovascular diseases and chronic kidney disease (CKD) (stage III or higher).

Immunological parameters included serum levels of IgG, IgA and IgM, measured both at diagnosis and at the most recent trough level. Lymphocyte counts, including total lymphocytes and subsets (CD3+, CD4+, CD8+, and CD19+), were recorded at diagnosis and after the initiation of IgRT. Data on actual or previous immunosuppressant therapy were systematically collected, covering current or previous use of corticosteroids, antimalarials, azathioprine, mycophenolate mofetil, methotrexate, cyclophosphamide, cyclosporine, tacrolimus, sirolimus, rituximab, belimumab, infliximab, etanercept, anakinra, tocilizumab, abatacept, and any other specified agents.

All clinical and relevant laboratory characteristics were compared across the two study groups (SCIg and IVIg) to identify potential differences in sociodemographics, infectious burden, comorbidities, organ involvement, laboratory profiles, and immunosuppressant treatment. Additionally, demographic characteristics and infectious and non-infectious complications were compared between 20% SCIg and facilitated SCIg formulations.

### 2.2 Statistics

The cross-sectional design of the study warranted a primarily descriptive analysis. Quantitative variables were presented as means and standard deviations, while categorical variables were expressed as absolute counts and percentages, excluding missing values. Clinical and immunological characteristics were compared across groups using the χ² test or Fisher’s exact test for categorical data, and the Student’s t-test for continuous variables after verifying normality assumptions. Statistical significance was defined as a p-value <0.05. All analyses and graphical representations were performed using R statistical software (version 4.4.1; 12).

### 2.3 Ethical considerations

The study was approved by the Ethics Committee of the University and Polytechnic Hospital La Fe (registration code: GIC-GAM-2020-01) and authorized by the Spanish Agency for Medicines and Health Products (AEMPS) under the same protocol. Each participating center independently obtained approval from their respective Institutional Clinical Research Ethics Committees. Patient anonymity and data confidentiality were maintained following Spanish regulations for observational studies. The study adhered to the Declaration of Helsinki and followed STROBE guidelines for reporting observational research.

## 3 Results

### 3.1 Cohort demographics and access to IgRT modalities

A total of 242 patients aged ≥16 years with a confirmed diagnosis of CVID, according to the ESID criteria (11), were included in the GTEM-SEMI-CVID Registry. The cohort comprised 118 males (48.8%) and 124 females (51.2%), with a mean age of 51.0 years (SD ±18.5), ranging from 17 to 94 years.

Two hundred and twelve patients were finally included in this study. Regarding IgRT, 124 patients (51.2%) received IVIg, 88 (36.4%) received SCIg (**Table 1**), and data on treatment was not available on 30 patients (12.0%). These missing data were due to incomplete retrospective documentation across multiple centers. Among SCIg-treated patients, 43 (48.8%) received 10% formulations and 44 (50.0%) received 20% formulations, while information was unavailable for one patient (1.2%).

**Table 1:** Demographic and IgG levels of patients receiving IgRT.

	IVIg (n=124)	SCIg (n=88)	p-value
Sex			
Male	59 (47.6%)	45 (51.1%)	0.676
Female	65 (52.4%)	43 (48.9%)	
Age	54.84 ( ± 18.58)	47.49 (± 16.61)	0.030
Age at diagnosis	43.12 (± 20.10)	37.85 (± 18.78)	0.055
Age at onset	30.63 ( ± 20.16)	27.75 (± 19.07)	0.313
Diagnosis delay (years)	11.15 ( ± 14.55)	9.85 (± 12.10)	0.517
IgG at diagnosis (mg/dl)	380.26 (± 234.93)	378.71 (± 188.20)	1
IgG trough levels on treatment (mg/dL)	838.88 (± 238.11)	899.04 (± 247.38)	0.43

SCIg, subcutaneous immunoglobulin; IVIg, intravenous immunoglobulin.

The analysis of IgRT modality according to the level of care of the participating hospitals revealed significant differences. In secondary hospitals, the majority of patients (82.6%) received IVIg, while only 17.4% were treated with SCIg. Conversely, tertiary hospitals demonstrated a more balanced distribution, with 55.6% of patients receiving IVIg and 44.4% treated with SCIg (p = 0.0136) (**Figure 1**).

A detailed analysis of patient age at the time of study inclusion showed a mean age of 54.8 years (SD ±18.6) in the IVIg group and 47.5 years (SD ±16.6) in the SCIg group, with a statistically significant difference (p = 0.003). The median age was 54 years (IQR 40–70.5) for IVIg patients and 43.5 years (IQR 35.8–60.3) for SCIg patients, with maximum ages of 94 and 86 years, respectively. Regarding age at diagnosis, the mean age was 43.1 years (SD ±20.1) in the IVIg group and 37.9 years (SD ±18.8) in the SCIg group, with median ages of 42 years (IQR 28–59.3) and 37 years (IQR 25–51), respectively. Maximum ages at diagnosis were 92 years for IVIg and 82 years for SCIg. No statistically significant difference was observed in the age at diagnosis between the two groups (p = 0.055) (**Table 1**).

Sex distribution was comparable between the IVIg and SCIg groups, with no statistically significant difference (p = 0.676). Among IVIg-treated patients, 59 (47.6%) were male and 65 (52.4%) were female, while the SCIg group included 45 (51.1%) males and 43 (48.9%) females (**Table 1**).

### 3.2 IgRT administration routes, dosage, and scheduling

Baseline IgG levels at diagnosis showed a mean of 379.4 mg/dL (SD ±215.9) across the cohort. Following immunoglobulin therapy, the mean IgG trough level increased to 866.5 mg/dL (SD ±242.7), based on available data from 40 and 59 patients, respectively. No significant differences were observed between IVIg and SCIg groups or between different SCIg formulations (**Tables 1**, **2**).

**Table 2:** Comparison of demographics, IgG levels, and comorbidities between 20% SCIg and facilitated 10% SCIg.

	20% SCIg (mean ± SD or n)	10% facilitated SCIg (mean ± SD or n)	p-value
Age (years)	45.47 ( ± 16.97)	49.60 ( ± 16.15)	0.245
Sex (Male/Female)	19 / 26	26 / 17	—
IgG at Diagnosis (mg/dL)	338.09 ( ± 132.85)	414.95 ( ± 222.12)	0.088
IgG Trough (mg/dL)	889.65 ( ± 243.19)	908.17 ( ± 254.59)	0.758
Weekly dose (g)	7.54 ( ± 2.54)	7.91 ( ± 2.64)	0.505
Infectious comorbidities			
Major bacterial disease	30 (66.7%)	23 (54.8%)	0.279
Opportunistic infection	5 (11.1%)	3 (7.1%)	0.715
Non-infectious comorbidities			
Cytopenia	16 (40.0%)	11 (26.2%)	0.241
Lymphadenopathies	14 (35.0%)	16 (38.1%)	0.821
Splenomegaly	17 (42.5%)	13 (30.9%)	0.360
Hepatic disease	7 (17.5%)	6 (14.3%)	0.654
Lung disease	28 (70.0%)	26 (61.9%)	0.491
Gastrointestinal involvement	19 (47.5%)	18 (42.9%)	0.825
Immune-mediated skin disease	12 (30.0%)	11 (26.2%)	0.807
Immune-mediated neurological disease	11 (27.5%)	3 (7.1%)	0.019
Systemic autoimmune disease	7 (17.5%)	8 (19.0%)	1.000
Solid or hematologic malignancy	4 (10.0%)	5 (11.9%)	1.000

SCIg, subcutaneous immunoglobulin.

The mean weekly immunoglobulin dose across the cohort was 7.4 g/week (SD ±2.6). The weekly dose ranged from 2.5 to 13.33 g/week for IVIg and from 3.0 to 13.33 g/week for SCIg. The median weekly doses were 6.8 g/week for IVIg and 8.0 g/week for SCIg. The mean doses were 7.2 g/week (SD ±2.6) for IVIg and 7.7 g/week (SD ±2.6) for SCIg. No statistically significant differences were observed between IVIg and SCIg groups (p = 0.142) No significant differences in weekly dose were observed between 20% SCIg and facilitated 10% SCIg (**Table 2**).

The most common administration schedule was monthly, applied to 106 patients (49.7%). Of these, 74.5% (n = 79) were treated with IVIg, while 25.5% (n = 27) received SCIg, specifically with 10% facilitated SCIg. The second most frequent schedule was triweekly administration, reported in 50 patients (23.4%). In this group, 64% (n = 32) were treated intravenously, and 30% (n = 15) received 10% facilitated SCIg. Data were unavailable for the remaining three patients.

A biweekly regimen was used in 24 patients (11.2%), with 70.8% (n = 17) receiving IVIg and 26.1% (n = 6) receiving 20% SCIg. Information was missing for one patient in this category. Weekly administration was followed by 27 patients (12.6%), all of whom were treated with 20% SCIg. Finally, six patients received alternative administration schedules that did not fit the primary regimens described.

A significant difference in the site of administration was observed between IVIg and SCIg groups (p = 1.03 × 10^-41^). Nearly all patients receiving IVIg (n = 122) were treated exclusively in a hospital setting, with only two patients receiving home-based therapy (1.6%). In contrast, the majority of SCIg-treated patients (80.6%, n = 71) administered their therapy at home, while 13.6% (n = 12) were treated in a hospital setting, and 5.7% (n = 5) alternated between both.

### 3.3 Infectious complications

Infectious complications did not differ significantly between groups. Major bacterial infections occurred in 68.5% of IVIg-treated patients and 60.2% of those on SCIg (p = 0.24). Opportunistic infections were observed in 11.3% and 9.2%, respectively (p = 0.66). No significant differences were found between 20% and facilitated 10% SCIg (**Table 2**).

### 3.4 Non-infectious comorbidities

Non-infectious comorbidities and complications were compared between the IVIg and SCIg groups. No significant differences were observed between the two groups in the prevalence of autoimmune diseases, immune-mediated cytopenias, lymphadenopathy, splenomegaly, pulmonary involvement (including GLILD and bronchiectasis), enteropathy, hepatic disease, immune-mediated skin or neurological affectation (**Table 3**). The prevalence of both solid and hematological malignancies was 17.7% in patients receiving IVIg (22 of 124) and 10.2% in those treated with SCIg (9 of 88). Although there was a numerical difference between the groups, it was not statistically significant (p = 0.17). Moreover, no significant differences in the presence of non-infectious complications were observed between 20% SCIg and facilitated 10% SCIg (**Table 2**).

**Table 3:** Non-infectious complications of patients receiving IgRT.

	IVIg (n = 124)	SCIg (n = 88)	p-value
Systemic autoimmune disease	26 (21.0%)	16 (18.2%)	0.840
Immune-mediated cytopenia	45 (36.3%)	28 (32.2%)	0.560
Lymphadenopathy	46 (37.1%)	30 (34.1%)	0.670
Splenomegaly	44 (35.5%)	31 (35.2%)	1.000
Lung disease	76 (61.8%)	58 (65.9%)	0.670
GLILD	7 (6.4%)	10 (12.8%)	0.260
Bronchiectasis	39 (31.5%)	36 (40.9%)	0.130
Non-infectious enteropathy	55 (44.4%)	37 (42.0%)	0.780
Diarrhea	43 (34.7%)	33 (38.4%)	0.660
Malabsorption syndrome	19 (15.4%)	14 (16.3%)	1.000
Hepatomegaly	29 (23.4%)	14 (15.9%)	0.163
Portal hypertension	13 (10.5%)	4 (4.5%)	0.345
Immune-mediated skin disease	35 (28.2%)	25 (28.4%)	1.000
Immune-mediated neurological affectation	13 (10.5%)	15 (17.0%)	0.217

GLILD, granulomatous-lymphocytic interstitial lung disease; IVIg, intravenous immunoglobulin; SCIg, subcutaneous immunoglobulin.

Cardiovascular risk factors were compared across the two treatment groups. Hypertension was observed in 4.9% of IVIg-treated patients (6 of 122) and 2.4% of SCIg-treated patients (2 of 83), with no significant difference (p = 0.37). Dyslipidemia was more frequent in the IVIg group (21.8%, 27 of 124) compared to the SCIg group (10.2%, 9 of 88), reaching statistical significance (p = 0.04). The prevalence of type 2 diabetes mellitus was similar between groups, affecting 8.1% of IVIg patients (10 of 124) and 9.1% of SCIg patients (8 of 88) (p = 0.91).

The presence of established cardiovascular damage, including heart failure, chronic cerebrovascular disease, and CKD was also evaluated. Heart failure was reported in 8.1% of IVIg-treated patients (10 of 124) and 6.8% of those treated with SCIg (6 of 88), without significant differences (p = 0.798). Cerebrovascular disease was noted in 2.4% of IVIg patients (3 of 124), while no cases were reported in the SCIg group (p = 0.27). CKD (stage III or higher) was present in 5.6% of patients receiving IVIg (7 of 124) and 3.4% of those treated with SCIg (3 of 88), with no statistically significant differences (p = 0.528).

Immunosuppressant treatment was documented in 41% of CVID patients treated with IVIg (50 of 122) and in 41.37% of those receiving SCIg (36 of 87), with no significant differences between the two groups (p = 1).

## 4 Discussion

This study provides a comprehensive analysis of IgRT patterns in a large cohort of Spanish patients with CVID, uncovering key differences between IVIg and SCIg in real-world clinical practice. The findings reveal: (i) a marked increase in SCIg adoption over the past decade, although IVIg remains the predominant modality, particularly in secondary hospitals where its use reaches 80.8%, in contrast to a more balanced distribution observed in tertiary centers; (ii) significant age-related differences influencing clinical decision-making, with IVIg being preferred for older patients and SCIg for younger ones; and (iii) neither the severity of hypogammaglobulinemia at diagnosis nor the overall clinical severity have a significant association with the received IgRT modality. These insights provide updated real-world evidence on IgRT utilization in Spain, highlighting the need to optimize therapeutic strategies to enhance patient outcomes and promote greater autonomy.

IgRT use has steadily increased in Spanish hospitals, driven by strong evidence for treating IEI (1) and expanding indications in autoimmune and neuromuscular diseases (13). IgRT is also widely used to prevent infections in SID driven by emerging therapies for hematological malignancies, which often induce profound and prolonged hypogammaglobulinemia (14). Despite this growing demand, updated national data on Ig usage have been lacking since the 2014 European Immunoglobulin Map (6). Our study addresses this gap, providing real-world data from 212 CVID patients across 15 Spanish hospitals. Although the GTEM-SEMI registry primarily aimed to assess clinical characteristics (10), it offers valuable insights into current IgRT practices and regional variations. The representation of both tertiary and secondary-level centers ensures a diverse spectrum of clinical settings, enhancing the generalizability of our findings.

Given the rarity and heterogeneity of CVID, a cohort of 212 patients is substantial and comparable to other national cohorts reported in neighboring countries, such as the Italian (224 patients; 15), American (205 patients; 16), British (334 patients; 17), and German cohorts (303 patients; 18). Of the 212 patients receiving IgRT, the majority were treated with IVIg over SCIg, highlighting the predominance of hospital-based IVIg as the main mode of administration for IEI patients in our setting, in accordance with other Mediterranean countries. Nevertheless, a significant shift towards SCIg use in PID patients has been observed in Spain, increasing from 15% reported in 2014 (6) to 41.9% in our study. Although still below the levels observed in Scandinavian countries—where SCIg has historically been preferred over IVIg since its early availability—this increase reflects a substantial change in clinical practice over the last decade in our national context. This change may reflect improved training of healthcare professionals and nursing units, greater patient autonomy, and recurrent shortages of plasma-derived products, prompting diversification of administration practices traditionally dominated by IVIg (19, 20).

Therefore, although the use of SCIg has increased notably in recent years, its broader implementation in Spain is hindered by the decentralization of the healthcare system, which is governed by autonomous communities and lacks unified national protocols. This decentralization leads to significant regional variability in access to SCIg, particularly between tertiary hospitals—where specialized units, structured care pathways, and trained personnel are more common—and secondary centers, where immunodeficiency care is typically managed within the scope of general internal medicine. Our study reflects this reality, with a more limited uptake of SCIg in secondary hospitals, where resources such as trained nursing staff, dedicated outpatient infrastructure, and experience with subcutaneous administration may be insufficient, and thus, SCIg possibility may not be offered to the patient (21).

In addition to these structural disparities, several systemic barriers contribute to the uneven adoption of SCIg. These include the lack of formal recognition for specialized nursing roles in immunodeficiencies, the absence of subspecialization pathways within internal medicine, and the chronic saturation of the public health system, which hampers the development of dedicated training programs for both physicians and nurses. Clinical inertia may further reinforce this imbalance, as IVIg is deeply rooted in internal medicine practice due to its long-standing use in autoimmune diseases. Consequently, IVIg often remains the default option, despite robust evidence supporting the safety, patient autonomy, and quality-of-life improvements associated with SCIg (22, 23).

Addressing this multifactorial gap will require coordinated efforts to enhance clinician training, strengthen infrastructure, and promote equitable access to SCIg. Additionally, leveraging digital tools such as national platforms, mobile apps, and educational content via social media offers a promising yet underused strategy (24). These technologies could help inform patients, support self-administration, and reduce disparities. Future efforts should consider integrating such tools into routine care in collaboration with professional societies and patient organizations. Importantly, all initiatives should also be tailored to the demographic reality of the target population.

In this work, the mean patient age exceeded 50 years old, likely reflecting the typical profile of patients managed in internal medicine departments, where older age and higher comorbidity burdens are common. Notably, IVIg-treated patients were approximately seven years older than those receiving SCIg. Despite being relatively younger, the average age of SCIg-treated patients in our study was still considerably higher than that reported in pivotal trials: 20.6 years in Japan, 34.4 years in the U.S., and 22.6 years in Europe (25). These findings suggest that, in real-world settings, SCIg is administered to older populations compared to those typically included in controlled clinical studies.

Our results are consistent with recent real-life studies indicating that advanced age does not preclude the use of SCIg. Observational studies have reported mean ages of 41.2 years (26), as well as cohorts exceeding 70 years, demonstrating safe home-based administration with various SCIg formulations, including 10% and 20% concentrations (27, 28). Similarly, other studies have shown high mean ages, such as 54.3 years for 10% SCIg, with 66.4% of patients being over 50 years old (29). These real-world data, in alignment with our results, challenge the perception that age is a barrier to SCIg self-administration, supporting its safe and effective use in older populations. Our findings advocate for broadening SCIg eligibility criteria to include elderly patients, recognizing its feasibility and clinical benefits.

Infection rates were comparable between IVIg and SCIg groups, with no significant differences in major bacterial infections, recurrent mild infections, or opportunistic infections. These findings align with a 2019 meta-analysis that reported no significant differences in overall or severe infection rates between both modalities (30). Non-infectious comorbidities, including autoimmune cytopenias, GLILD, inflammatory enteropathies, lymphoproliferation, bronchiectasis, and neoplasms, were common in our cohort but did not differ significantly between IVIg and SCIg groups, suggesting they do not influence the choice of IgRT route. Dyslipidemia was significantly more frequent in IVIg-treated patients (22% *vs*. 10%). However, its clinical relevance remains unclear and may reflect the older age of this group, although an association with CVID has been previously reported (31).

In our cohort, the mean weekly IgRT dose was comparable between modalities, with 7.16 g for IVIg and 7.72 g for SCIg, showing no significant differences and aligning with previous real-world data. Studies such as Gathmann et al. (9) and the FIGARO study (26) reported similar weekly doses of approximately 7.5–8 g in adult patients, while a German study noted slightly lower doses for 20% SCIg (~6.5 g/week) (32). Post-treatment IgG trough levels were also similar between groups, with SCIg-treated patients showing slightly higher levels (899 mg/dL) compared to IVIg (839 mg/dL), consistent with Shrestha et al. (30), which highlighted more stable IgG levels with SCIg. Recent pharmacokinetic studies have further demonstrated that SCIg, particularly at 20% concentrations, achieves stable long-term IgG trough levels and reaches target levels more rapidly than IVIg, making it a suitable option for patients with severe and profound hypogammaglobulinemia (33). Notably, in our cohort, neither severe hypogammaglobulinemia nor a more severe clinical phenotype was associated with a higher use of IVIg, suggesting that clinical severity does not drive the choice of IVIg over SCIg. This finding reflects an appropriate therapeutic approach, indicating that SCIg is being utilized effectively even in patients with severe immunodeficiency profiles.

Regarding administration frequency, monthly dosing was predominant, followed by every-three-week schedules. Weekly and biweekly regimens were less common, reflecting the lower use of 20% SCIg in our cohort. These patterns are in line with real-world studies where facilitated 10% SCIg is often administered every four weeks, while 20% SCIg tends to follow weekly schedules (26, 29, 32).

IVIg was predominantly administered in hospital settings (122 patients), with home treatment being rare (2 patients), reflecting the traditional practice in Spain. In contrast, 80.6% of SCIg-treated patients self-administered at home, while 13.6% received it in hospitals and 5.7% in both settings. Real-world studies confirm that 20% SCIg is mostly managed at home (27, 32), while 10% facilitated SCIg is often hospital-based: 37.4% in the U.S. (29) and up to 41.1% among older patients in Europe (26). These findings suggest that 10% facilitated SCIg may serve as a hospital-based alternative to IVIg for patients with poor venous access or limited autonomy.

This study has several limitations. Its cross-sectional and retrospective design precludes causal inferences and limits the assessment of temporal dynamics, including potential switches between IgRT modalities over time. Additionally, this design may contribute to incomplete or missing data for certain variables. The GTEM-SEMI-CVID Registry is restricted to Spanish centers, potentially limiting the generalizability of findings to other healthcare settings. The predominance of hospital-based IVIg in secondary centers and the limited access to SCIg may reflect regional disparities rather than purely clinical indications. Additionally, confounding factors such as socioeconomic status, patient preferences, and logistical barriers were not evaluated. The lack of systematic data on adverse events, patient-reported outcomes, and long-term follow-up restricts the analysis of clinical outcomes, including infection rates and organ damage progression. Furthermore, patient weight was not consistently documented across all cases, which prevented standardized calculation and reporting of weight-adjusted IgRT doses in mg/kg/week or mg/kg/month. Future prospective studies with broader geographic representation and patient-centered data are needed to address these limitations.

## 5 Conclusion

This study provides a comprehensive overview of real-world IgRT practices in Spanish patients with CVID, highlighting significant differences between IVIg and SCIg usage across tertiary and secondary hospitals. While IVIg remains the predominant modality, especially in secondary centers, SCIg use has markedly increased in recent years, reflecting shifts towards greater patient autonomy and home-based care. Our results suggest that IgRT route selection is shaped less by immunological or clinical severity than by contextual factors, including institutional infrastructure, regional healthcare disparities, and availability of trained personnel. Notably, SCIg was safely used in older populations, challenging age-related prescribing biases. Addressing disparities in access, particularly in secondary hospitals, may enhance equitable use of SCIg and optimize patient outcomes. In this regard, digital tools and mobile health technologies may offer additional opportunities to support patient education, self-administration, and equitable access to SCIg. Future longitudinal studies are needed to validate these findings and explore the long-term clinical benefits of both modalities in diverse healthcare settings.

## Data Availability

The original contributions presented in the study are included in the article/supplementary material. Further inquiries can be directed to the corresponding authors.
